# Intron Retention and Alzheimer’s Disease (AD): A Review of Regulation Genes Implicated in AD

**DOI:** 10.3390/genes16070782

**Published:** 2025-06-30

**Authors:** Ayman El-Seedy, Véronique Ladevèze

**Affiliations:** 1 Laboratory of Cellular and Molecular Genetics, Department of Genetics, Alexandria University, Aflaton Street, El-Shatby, Alexandria 21545, Egypt; ayman.el-seedy@alexu.edu.eg; 2 Laboratoire MOVE-UR20296, University of Poitiers, Pôle Biologie Santé-Bât B36, 1 rue G. Bonnet-TSA 51156, Cedex 9, 86073 Poitiers, France

**Keywords:** intron retention, alternative splicing, Alzheimer’s disease (AD), aging, AD-associated genes, a potential diagnostic biomarker

## Abstract

Determining the genetic variations of candidate genes in affected subjects will help identify early pathological biomarkers of Alzheimer’s disease (AD) and develop effective treatments. It has recently been found that some genes that are linked share an increase in intron retention (IR). In this review, we discuss a few instances of mRNA-IR in various genes linked to AD, including *APOE*, *MAPT-Tau*, *Psen2*, *Farp1*, *Gpx4*, *Clu*, *HDAC4*, *Slc16a3*, and *App* genes. These genes are vulnerable to IR, encompassing additional crucial proteins for brain functionality, but they are frequently involved in pathways linked to the control of mRNA and protein homeostasis. Despite the advancements in human in vivo RNA therapy, as far as we know, there are no reports of data generated regarding artificial in vivo splicing in either animal models or humans. To prevent genetic variations and improve or repair errors in expression of desired genes, humans have adopted new gene editing techniques like CRISPR-Cas9 and RNAi modalities. Ultimately, IR could be utilized as a therapeutic potential biomarker for disorders related to intronic expansion.

## 1. Introduction

Alzheimer’s disease (AD) is defined by slow and gradual neurodegeneration induced by the death of neuronal cells. The entorhinal cortex of the hippocampal region is usually where the neurodegenerative process starts. Early- and late-onset AD have been linked to genetic variables [1]. It is the leading cause of neurodegenerative disorders among elderly individuals. Clinically, individuals first show signs of short-term memory impairment, later experiencing executive dysfunction, confusion, agitation, and behavioral issues [2]. The development of Alzheimer’s disease is influenced by a variety of genes and environmental factors, making it a complex, multigenic disorder. These consist of, but are not restricted to, *APOE*, *Tau-MAPT-Psen2*, *Farp1*, *Gpx4*, *Clu*, *HDAC4*, *Slc16a3*, and *App* genes.

Alternative splicing (AS) has been known in humans for many years, yet intron retention (IR), a type of AS, was initially reported in non-mammalian organisms like plants, fungi, insects, and others. IR is defined by the retention of one or more intron(s) in the final mRNA transcripts (mRNA-IR). This retention frequently triggers the formation of premature termination codons (PTCs) and, thus, mRNA-IR degradation by nonsense-mediated decay (NMD) [3]. Therefore, mRNA-IR is difficult to detect and quantify. If this NMD mechanism is well known, we could suggest other hypotheses, such as the translation of IR transcripts produce alternative proteins with novel functions, or, as the IR transcripts stay in the nucleus, they could be degraded by an independent mechanism different to NMD. Moreover, the storage of mRNA-IR in the nucleus could be quickly exported to the cytoplasm upon specific stimuli with, or without, introns in their sequence. If mRNA-IR was cleaved in cytoplasm, another hypothesis is to create miRNA [4].

In mammalians, the appearance of the intron retention gene transcript in the brain cells could be a sign of cellular stress and contribute to its detection with progressive aging and Alzheimer’s disease [5,6,7,8,9]. Some tissues use intron retention more than others. This is the case for neurons, which rapidly produce proteins in synaptic plasticity patients and age-sex matched controls [10,11,12,13]. These observations were confirmed in the hippocampus [14]. The first study showing a significant increase in intron retention in the frontal cortex was realized by Twine et al. [15], and characterized the genes implicated in homeostasis and synaptogenesis. RNA-seq analysis was obtained on postmortem cortical and hippocampal samples of Alzheimer’s patients [16]. To detect IR, bioinformatics tools combine alignments splice-aware (vias STAR) and quantification tools (IRFinder) with a standardization taking into account the sequencing and the length of introns [17].

Moreover, a new category of introns called detained introns (DIs) has been identified: these DIs stay in the nucleus and are shielded from degradation, causing a slower splicing rate compared to other introns in the same gene [13,18,19]. This mechanism explains that mRNA-IRs could be translocated to the cytoplasm, and they are named Cytoplasmic Intron Retaining Transcripts (CIRTs) [20,21]. Due to the presence of PTC, these CIRTs may be broken down in the cytoplasm by the mRNA surveillance pathways [22], as well as NMD [23,24]. GC content is higher in retained introns than in normal spliced introns [5,25,26]. The interaction of genetic and epigenetic elements, such as DNA methylation, may control IR.

Additionally, it has been proposed that detained introns (DIs) influence the degree of gene expression, as shown by Boutz et al. [18]. They used a comprehensive sequencing technique of embryonic stem cell RNA to identify numerous distinct internal introns that are noticeably more prevalent than the other introns within polyadenylated transcripts. In addition to that, a number of DIs expressed in humans, mouse cell lines, and in the livers of adult mice are evolutionarily preserved [18]. In the human transcriptome, retained introns contain a high GC content [27].

The survey of IR-mRNAs from cellular regulatory control becomes increasingly evident through deep sequencing [28]. Moreover, the biological role of IR-mRNAs in both physiological and pathological states seems to be highly important owing to the advancement of computational analyses [29,30]. Recently, in mammals, intron retention was described in cancers and other diseases as Alzheimer’s disease (AD) or during aging. The implication of some IR in AD or aging could have biological relevance for the regulation of different mechanisms.

IR might play different roles in regulating the development of animal and the aging process. For instance, there is an increased number of retained introns in the young 2-week-old mice in the frontal cortex, whereas it is subsequently lost in the older animals. Many of these genes have biological functions specific to cell cycles, epigenetic regulation, and brain development. According to different authors [13,25,31], IR frequently plays a role in controlling neurogenesis. The homeostasis of mRNA and protein molecules is regulated by genes with an increased level of retained introns in both aged and diseased mammalian brain tissues [5]. This suggests that abnormal patterns of intron retention might contribute to the decline of proteostasis, a key characteristic of aging and Alzheimer’s disease etiology. IR’s possible regulatory effect on mRNA processing could also serve as a feedback loop to raise IR levels in the brains of AD patients or elderly animals [5]. Middleton et al. [17] described that IR accounts for approximately 80% of genes that code for proteins in hundreds of human coding genes, and is associated with cell cycle and differentiation. However, IR was significantly associated with lower protein levels. When genes exhibit differential IR, several common characteristics emerge, including reduced intron length, stable mRNA levels, and an abundance of biological functions linked to mRNA processing and proteostasis [5]. In older adults, differential IR occurs in multiple AD-associated genes and impacts different biological functions at different ages.

NMD commonly breaks down mRNA transcripts with retained introns to eliminate mRNAs whose expression is not necessary for the operation of particular cell types [25]. The expression of genes that are functionally related is also regulated by a similar mechanism during granulocyte differentiation [26]. IR, however, can also serve as a tactic to enable the quick mobilization of particular mRNAs for the translation of proteins in response to particular developmental cues. Mouse neocortical cells showing nuclear accumulation of these transcripts with retained introns serve as one illustration [13].

It was also demonstrated by the authors of [5] that a post-transcriptional signature linked to AD and progressive aging is increased intron retention. The GC content of the differentially retained introns found in the AD frontal cortex is higher, and many of their transcripts exhibit different levels of protein expression to those found in control samples. Their findings imply that an elevated IR is a conserved signature linked to aging. The degree of protein expression may be impacted by differential IR as retained introns in the transcript may cause mutations or premature termination during translation. In late-onset sporadic AD, alterations in the IR pattern with aging may control the shift from a healthy to a pathological state by influencing pathways related to mRNA and protein homeostasis.

They demonstrate a significant decrease in the expression of 80 protein isoforms encoded by 73 differential IR genes, while 41 IR genes exhibit an increase in expression. This suggests that differential IR contributes to the altered proteome seen in the frontal cortex of AD. For example, there were significant correlations of intron expression within innate immune genes, like HMBOX1, with AD in humans [32].

Furthermore, according to [5], in AD samples, the rate of IR in genes involved in RNA processing and protein homeostasis is significantly higher, and that IR often causes notable changes in their protein levels in comparison to age-matched controls. According to these findings, elevated IR is a transcriptional signature of aging and could be connected to the development of AD. In AD tissues, over 1100 more IR events are occurring in about 780 genes than in the healthy frontal cortex. Given that mRNA expression seems unaffected, IR does not cause NMD. Nevertheless, approximately 15% of these elevated IR genes exhibit notable variations in their protein levels between the AD frontal cortex and a healthy control, indicating that the abnormal proteomic landscape in the AD frontal cortex might be influenced by the translation of mRNA-IR transcripts [33]. In addition, notable variations in IR levels in numerous genes were detected between AD and their age-matched control tissues [5]. Between age-matched control and AD tissues, over 3800 differential IR events were found in the cerebellum. The AD cerebellum exhibited elevated IR in about 80% of these differential IR events.

## 2. Methods

An extensive literature search was performed utilizing the PubMed, Web of Science, and Scopus databases. Keywords pertaining to genes associated with Alzheimer’s disease and alternative splicing along with intron retention were utilized. The investigation focused on articles released from January 2000 through January 2025. Inclusion criteria included studies that were published in English and provided original data. Criteria for exclusion included studies that did not address any review questions. A two-step screening method was utilized, initially examining titles and abstracts, followed by full texts of possibly relevant articles. Data extraction centered on intron retention associated with AD. The search strategy was intended to be thorough but recognizes possible constraints related to database coverage and the specificity of search terms.

## 3. Results and Discussion

**Figure 1:**
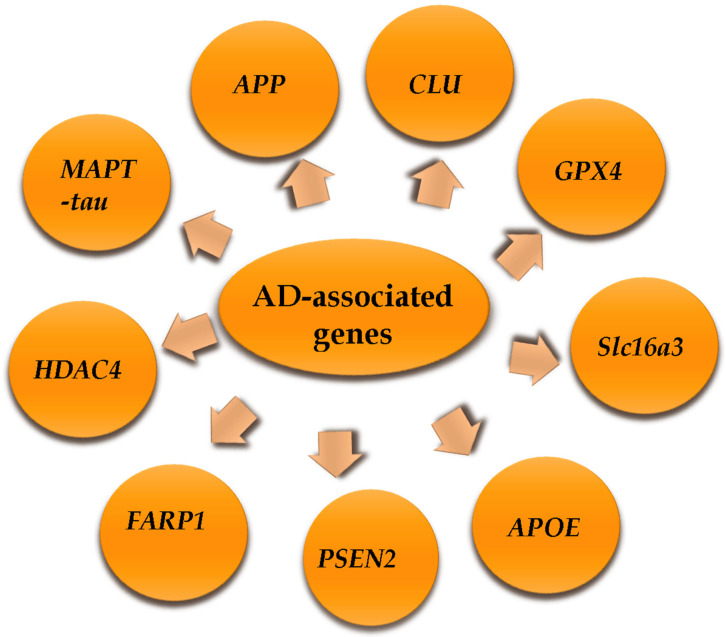
Genes associated with increased or decreased risk for Alzheimer’s disease in human.

Determining the genetic variations of candidate genes in affected subjects will help identify early pathological biomarkers of Alzheimer’s disease (AD) and develop effective treatments. It has recently been found that some linked genes share an increase in intron retention (IR). In this review, we describe some examples of mRNA-IR in genes influencing Alzheimer’s disease in humans and mice and discuss a few instances of mRNA-IR in various genes linked to AD, including APOE, MAPT-Tau, PSEN2, FARP1, GPX4, CLU, HDAC4, Slc16a3, and APP genes (Figure 1 and Table 1). These genes are vulnerable to IR and include other important proteins for brain function, but they are frequently involved in pathways linked to the control of mRNA and protein homeostasis.

### 3.1. Types of AD-Associated Genes

#### 3.1.1. Apolipoprotein E (*APOE*) Gene

APOE variants are the primary genetic contributors to late-onset Alzheimer’s disease (LOAD). The *APOE*4 allele confers increased risk (around 15% frequency in the general population and at 50% in LOAD [43,44]. *APOE* contains 4 exons and 3 introns located on 19q13.32, encoding a total of 317 amino acids (aa). The mature APOE protein consists of 299 amino acids following the removal of the signal peptide. The translation initiates in exon 2 and terminates in exon 4. APOE is primarily secreted by astrocytes [45,46,47] with microglia [48]. An APOE isoform with intron 3 (APOE-I3) was detected, and this variant was restricted to neurons and increased with neuronal stress in murine models [49]. APOE-I3 encodes a truncated 79aa, due to a PTC, suggesting a candidate for NMD. However, APOE-I3 seems to be degraded slowly, but the role of this isoform in the brain is unclear [37]. These authors insist that this mRNA-I3 is rare. Moreover, the minor C allele of rs12982192 (this single-nucleotide polymorphism (SNP) is on the same haplotype as APOE4) appears associated with increased APOE-I3, suggesting a modulation of IR to the association of APOE4 (the primary genetic risk factor for AD.

More recently, Chen et al. [50] showed that this APOE-I3 was more abundant in AD with more severe tau and amyloid pathological burden. These authors showed a dosage-dependent increase in the intron retention event with the APOE-ε4 allele. Although the function of intron-3 retention was not elucidated, their results are in line with earlier research that showed that IR events generally increase with age and AD, with implications for post-transcriptional regulation [5].

#### 3.1.2. Microtubule-Associated Protein Tau (*MAPT-Tau*) Gene

Abnormal tau inclusions are a hallmark of a subset of neurodegenerative diseases known as tauopathies [51]. Exons 0 and 1 encode the 5′ UTR of MAPT mRNA, while exon 14 encodes the 3′ UTR. The microtubule-associated protein tau (MAPT) gene, which is found on chromosome 17q21.31, is the genetic source of tau protein. Only the mRNA of peripheral tissue contains tau, as exons 4a, 6a, and 8 are skipped during brain transcription [52]. There are six distinct tau isoforms in the central nervous system (CNS) that vary in length from 352 to 441 residues due to alternative splicing of exons 2, 3, and 10 [53,54,55].

Tauopathies like neurodegenerative frontotemporal dementias and AD may exhibit deviations from the equimolar amounts of 4R- and 3R-tau found in a healthy adult brain [56,57]. Furthermore, N-ter tau truncation (20–22 kDa fragment NH2htau) is an important pathophysiological factor that contributes to the development and onset of AD [58]. Cultured neurons in the hippocampus exposed to low concentrations of extracellular A-oligomers showed this fragment [59].

Spliceosome activity is disrupted in AD by Tau neurofibrillary tangle pathology. The transcriptome disruption caused by RNA-splicing errors, such as IR and non-annotated cryptic junctions, is linked to Tau-mediated neurodegenerative processes [60].

Intron 12 retention gives rise to a new tau isoform called W-tau [61]. This tau isoform causes a PTC, which is followed by a canonical polyadenylation sequence that truncates the protein. This isoform is, therefore, distinct from other human tau isoforms in that it lacks exon 13 of the MAPT gene and has an 18-amino acid sequence at its carboxyl-terminal region that corresponds to the translation of the retained fragment of intron 12 in its place. AD reduces this novel non-aggregative splicing isoform. Tryptophan (W), an amino acid that is missing from the remainder of the human tau sequence, is present in two residues in the 18-residue sequence. When the beginning of intron 12 is retained and exon 13 is truncated, W-tau isoforms exhibit neuroprotective characteristics like a decreased capacity for aggregation or the capacity to prevent the polymerization of other tau isoforms. In vitro, the W-tau isoform inhibits the aggregation of Tau and amyloid beta peptide [62].

The cortexes of AD patients’ brains exhibit elevated levels of the tau11i isoform, which is produced by intron 11 retention, in addition to W-tau isoforms. Tau11i forms aggregates that weakly colocalize with 4R-tau fibril-like structure in the AD temporal lobe and is enriched in the sarkosyl-insoluble fraction in the AD hippocampal region. Furthermore, in human mature cortical neurons, Tau11i, which is stably expressed, exhibits weaker co-localization with α-tubulin of the microtubule network. Moreover, the protein Tau is responsible for the formation of neurofibrillary tangles observed in AD [63].

To explain these differences, Tri-dimensional structural analysis of Tau isoforms was realized by [64]. In the C-ter domain of W-Tau, the loss of the alpha helix is the main change. Moreover, Intron-12-retaining species are present even less prominently than those retaining intron-3 [65]. Moreover, some transcripts retain both introns 3 and 12 and contain tryptophan and another isoform of the W-Tau family. These intron-retaining species are diminished in brain samples of AD patients with respect to individuals without dementia [36]. mRNA-IR-3 does not generate a PTC because this addition does not cause a shift in the reading frame, suggesting a protein that could be triggered pre- and post-translational modifications. At the opposite, a PTC was present in mRNA-IR-12. Nevertheless, a canonical poly A site is located in the same intron afterward [66].

#### 3.1.3. Presenilin 2 (*Psen2*) Genes

Pathogenic variations in 1 (PSEN1), which is found on chromosome 14q24.2, and presenilin 2 (PSEN2), which is found on chromosome 1q42.13, contribute to early-onset familial Alzheimer’s disease (EOAD, onset < 65 years of age). Alzheimer’s disease, both sporadic and familial, has been linked to alternative splicing of these two genes. A human-specific cryptic exon in PSEN2’s intron 9 and a 77 bp intron retention product before exon 6 are two examples of alternative splicing patterns of PSEN2 unique to sporadic AD that are significantly elevated in sporadic Alzheimer’s disease samples. Additionally, the percentage of canonical full-length PSEN2 transcripts is significantly lower in sporadic AD samples compared to familial AD samples and controls [38]. The open reading frame would be restored by this IR, which would also produce a full-length PSEN2 with 25 addition aa [67].

Course et al. [38] counted the number of reads per sample with a transcript origin in PSEN2 (chr1:226,870,616-226,896,098). They identified frequent RNA editing at short interspersed nuclear elements and showed that PSEN2 contains Alu elements, specifically, in a long 3′ untranslated region. Furthermore, a lengthy 3′UTR of PSEN2 also contained long interspersed nuclear elements (LINEs). The knowledge of PSEN1 and PSEN2 variations in AD indicates that PSEN2 transcript variations may contribute to sporadic AD and points to new pathophysiological mechanisms.

#### 3.1.4. FERM, Rho/ArhGEF, and Pleckstrin Domain Protein 1 (*Farp*) Gene

Farp 1—FERM, ARH/RhoGEF, and pleckstrin domain protein 1 are the different names of this protein. *Farp1* gene is located on chromosome 13q32.2 and contains four exons. This gene has 42 transcripts due to splice variants. A lengthy intron of the *Farp1* gene was detected. IR may result in a novel isoform in mice (800aa) [32]. As Farp1 is involved in synapse formation [68], IR of Farp1 could be connected to synaptic activity. Nonetheless, the IR might be unrelated to AD as synapse function may be independent of AD

#### 3.1.5. Glutathione Peroxidase 4 (*Gpx4*) Gene

*Gpx4* is located in human chromosome 19p13.3. GPx4 consists of three isoenzymes located in the nucleus (n-GPx4), the endoplasmic reticulum, mitochondria (m-GPx4), and the cytoplasm (c-GPx4) of mammalian cells. Despite having different N-terminal sequences, all of these isoforms are fairly similar [69,70,71]. GPx4 isoenzymes are produced by alternative splicing from a single gene, Gpx4. There are eight exons in the Gpx4 gene, and exons 3–8 encode the same functional enzyme in all three isoforms. GpX4n could have an IR that is present only in Gpx4intron 1b. Docosahexaenoic acid (DHA) inhibits all splicing variations of the gene, including the intron-retaining mRNA, in a transgenic APP/PS1 model of AD. The extent of this effect, however, varies depending on the diet and isoform [39]. The lack of effect in animals fed DHA diets suggests that Gpx4 expression (and particularly the cytoplasmic intron sequence-retaining Transcripts isoform) is a very adaptable strategy to sustain membrane DHA in severe unfavorable conditions. This is because amyloid β accumulation (caused by overexpression of the APP and PS1 transgenes) and a lack of DHA supply to the brain are stressing conditions that reinforce Gpx4 expression [72].

#### 3.1.6. Clusterin (*Clu*) Gene

The *Clu* gene is located at human chromosome 8p21-p12 and contains nine exons. The CLU protein is an extracellular molecular chaperone and is overexpressed in several human neurodegenerative diseases such as AD [73]. Indeed, *Clu* is another risk gene associated with LOAD. More specifically, the CLUrs11136000 polymorphism is significantly associated with AD in Caucasian and Asian populations [74] as well as two other single-nucleotide polymorphisms (rs2279590, and rs9331888) [75]. CLU is regarded as a contributor to heightened amyloid dyshomeostasis linked to the initial female brain transition, offering a mechanistic explanation for the increased vulnerability of the female brain to AD. [76]. One SNP (rs7982) within a splicing regulatory element in the fifth exon plays a exon role on AS. Indeed, this SNP was significantly associated with IR. More interestingly, the IR rates decreased more significantly in AD females that in males. A sex-dependent role of rs7982 was suggested in AD via IR regulation [40]. Different techniques were used such as RNA sequencing (RNA-Seq) based on brain tissue and whole genome sequencing (WGS) to detect this SNP and its impact [41].

#### 3.1.7. Human Histone Deacetylase 4 (*HDAC4*) Gene

The *HDAC4* gene is located on chromosome 2q37.3. Mouse HDAC4 contains 31 exons and is located on chromosome 1. It encodes histone deacetylase 4, and causes the compaction of chromosomes. This gene has 18 splice transcripts. In D50 flies, a retained intron at the HDAC4 gene results in a PTC, leading to a shorter HDAC4 protein [5]. HDAC4, known for its role in deacetylating histones and nuclear factors, has been suggested to play a part in long-term memory formation in flies [77].

#### 3.1.8. Solute Carrier Family 16, Member 3 (*Slc16a3*) Gene

Both normal and pathological aging appear to be linked to a mechanism of intron retention (IR) for the MCT4 transcript (*Slc16a3* gene located on chromosome 17q25.3) that was found in the brain of mice. The APP/PS1 mouse model of Alzheimer’s disease is one example of a neurodegenerative disease in which this phenomenon is present, and its prevalence rises with age. Out of the six exons in this gene, only the intron 2 that lies between exons 2 and 3 could be kept. Since cytokine (IL-1β) mimicking an inflammatory signal can induce IR of the MCT4 transcript, astrocytes appear to be especially susceptible to this phenomenon [35]. A PTC appears in IR-mRNA, inducing the synthesis of a short protein. Moreover, Intron retention could be used as a molecular marker that differentiates melanoma from non-melanoma cancer cells in greek patient [34].

#### 3.1.9. Amyloid Beta Precursor Protein (*App*) Gene

*App* gene coding for the Amyloid Beta Precursor protein is localized on chromosome 21 (21q21.3 in mouse) and contains 19 exons [42]. In vivo therapeutic exon skipping is made possible by a very effective base editing toolbox. This toolbox allows for the simultaneous editing of splice acceptor (SA) and splice donor (SD) sequences by fusing to adenosine or cytosine deaminases. Enhanced exon skipping, decreased aberrant splicing, and the ability to skip exons that are resistant to single splice site editing are all made possible by synchronized SA and SD editing. They select APP exon 17, for instance, which codes for amino acids that are broken down to create Aα-42 plaques in AD brains [78].

In fact, exon skipping could induce splicing aberrations such as cryptic splicing and IR. Indeed, IR could occur when disruption of an SA or SD signal does not induce full exon skipping and so a part of an intron or the entire intron could be included into the mature mRNA [79,80]. In the AAP gene, if intron 17 is present in mature mRNA, it induces a PTC in the sequence.

## 4. Conclusions

Interestingly, it has been found that genome-wide splicing studies related to aging frequently show an increase in intron retention. The incidence of IR appears to increase with age in Drosophila melanogaster, Caenorhabditis elegans, Saccharomyces cerevisiae, mice, and humans [5,81]. Alzheimer’s disease (AD) may be seen as a hastened form of aging. Notably, IR was found in approximately ~780 genes within brain tissue affected by AD [5,82]. Widespread IR has the potential to influence gene expression and enhance transcriptome variability with phenotypes linked to senescence, significantly broadening the biological relevance of IR [9]. Genes vulnerable to IR are frequently involved in pathways that govern mRNA and protein balance, while also encompassing other essential proteins for brain activity.

It is important to note that IR has been increasingly recognized as an AS nuclear mRNA, introducing a new layer of complexity in the regulation of gene expression [82]. Valadkhan et al. [83] created a small spliceosome capable of effectively catalyzing intron removal in vitro by mimicking the initial two trans-esterification splicing reactions in the nucleus with the mammalian catalytic core of spliceosomes, U2 and U6 snRNA. To create IR-free RNA products, the results showed that introns could be effectively extracted from synthetic oligonucleotide constructs and then exon ligated [84,85].

As far as we are aware, although advancements have been achieved in conducting in vivo RNA therapies in humans, there are no documented cases of artificial in vivo splicing occurring in humans or in animal models. New methods for gene editing, such as RNAi modalities (ex. lipid, organic, or inorganic nanocarrier systems) and CRISPR-Cas9, have been utilized in humans to prevent gene mutation and enhance or rectify gene expression [86]. IR may have therapeutic uses as well as potential diagnostic biomarkers for intronic expansion disorders [87,88].

## Figures and Tables

**Table 1:** List of AD-associated genes influencing Alzheimer’s disease in humans and mice.

Gene	Location	Function	Retained Intron	Detection Techniques	Reference
*Slc16a3*	In humans, chromosome 17.	Encodes the Monocarboxylate transporter 4(MCT4).	Intron 2/3 in human melanoma samples (443 bp).	RT-sqPCR.	Giannopoulou et al., *Int. J. Mol. Sci.* 2019, *20*(4), 937 [34]
	In mice, chromosome 11.	Encodes the MCT4.	Intron 2.	RT-sqPCR.	El-Seedy et al., *Genes (Basel)*. 2023 17,14(10):1949 [35]
*MAPT*	In humans, chromosome 17.	Encodes the protein Tau.	Intron 3, intron 12 or both in human brain samples.	qRT-PCR.	Ruiz-Gabarre et al., *EBioMedicine* 2024, [36] Feb,100:104953. doi: 10.1016/j.ebiom.2023.104953.
*APOE*	In humans, chromosome 19.	Encodes the apolipoprotein E protein.	Intron 3.	qRT-PCR	Dieter & Estus, *Mol. Neurodegeneration* 2010, 5, 34 [37].
*PSEN2*	In humans, chromosome 1.	Encodes the presenilin-2 protein.	77 bp intron retention product before exon 6.	Long-read sequencing technology.	Course et al., *Brain.* 2023 13,146(2), 507–518. doi: 10.1093/brain/awac294. PMID: 35949106; [38]
*FARP1*	In humans, chromosome 3.	Encodes FERM, Rho/ArhGEF, and Pleckstrin domain protein 1.	Different introns in human and mouse brain with Alzheimer’s disease.	Nanostring chip and RNA-seq data.	Li et al., *Alzheimers Dement*. 2021, 17(6):984–1004 [32]
*GPX4*	In humans, chromosome 19.	Encodes for the glutathione peroxidase 4 enzyme.	In a hippocampal cell line, 213 bp sequence related to intron I1b situated between exons E1b and E2.	RT-qPCR.	Casañas-Sánchez et al., *Front Physiol.* 2015, 22;6:203 [39]
*CLU*	In humans, chromosome 8.	Encodes the protein clusterin.	The rs7982 variant is situated in the 5th exon of CLU gene, which splits this exon into two sections by a brief intron.	RNA sequencing (RNA-Seq) based on brain tissue and whole genome sequencing (WGS).	Han et al., *Int J Mol Sci*. 2020 25;21(19):7079 [40]
					Harold et al., *Nat. Genet.* 2009, 41:1088–1093 [41]
*HDAC4*	In humans, chromosome 2.	Encodes the Histone deacetylase 4, protein.	Varied introns in the prefrontal cortex (PFC) of young and older humans.	Mammalian RNA-seq datasets.	Adusumalli et al., *Aging Cell*. 2019, 18(3):e12928 [5]
*APP*	In humans, chromosome 21.	Encodes the amyloid precursor protein.	Introns 6, 7, and 8.	Brain tissue of hAPP transgenic and nontransgenic mice and of humans with and without AD.	Rockenstein et al., *Journal of Biological Chemistry*. 1995, 270, 47 (24), 28257–28267 [42]

## Data Availability

All presented data are available on request from the corresponding author.

## References

[1] Kumar A., Sidhu J., Lui F., Tsao J.W. (2025). Alzheimer Disease. StatPearls [Internet].

[2] Bekris L.M., Yu C.E., Bird T.D., Tsuang D.W. (2010). Genetics of Alzheimer disease. J. Geriatr. Psychiatry Neurol..

[3] Belgrader P., Cheng J., Zhou X., Stephenson L.S., Maquat L.E. (1994). Mammalian nonsense codons can be cis effectors of nuclear mRNA half-life. Mol. Cell. Biol..

[4] Monteuuis G., Wong J.L., Bailey C.G., Schmitz U., Rasko J.J. (2019). The changing paradigm of intron retention: Regulation, ramifications and recipes. Nucleic Acids Res..

[5] Adusumalli S., Ngian Z.K., Lin W.Q., Benoukraf T., Ong C.T. (2019). Increased intron retention is a post-transcriptional signature associated with progressive aging and Alzheimer’s disease. Aging Cell.

[6] Olthof A.M., Hyatt K.C., Kanadia R.N. (2019). Minor intron splicing revisited: Identification of new minor intron-containing genes and tissue-dependent retention and alternative splicing of minor introns. BMC Genom..

[7] Huang H.D., Horng J.T., Lin F.M., Chang Y.C., Huang C.C. (2005). SpliceInfo: An information repository for mRNA alternative splicing in human genome. Nucleic Acids Res..

[8] Qin N., Zhang S.P., Reitz T.L., Mei J.M., Flores C.M. (2005). Cloning, expression, and functional characterization of human cyclooxygenase-1 splicing variants: Evidence for intron 1 retention. J. Pharmacol. Exp. Ther..

[9] Yao J., Ding D., Li X., Shen T., Fu H., Zhong H., Wei G., Ni T. (2020). Prevalent intron retention fine-tunes gene expression and contributes to cellular senescence. Aging Cell.

[10] Li Q., Lee J.A., Black D.L. (2007). Neuronal regulation of alternative pre-mRNA splicing. Nat. Rev..

[11] Norris A.D., Calarco J.A. (2012). Emerging Roles of Alternative Pre-mRNA Splicing Regulation inNeuronal Development and Function. Front. Neurosci..

[12] Raj B., Cedillo J., Zhang K. (2014). A high-thrοughput screen οf intrοn retentiοn in neurοnal transcripts. RNA.

[13] Mauger O., Lemoine F., Scheiffele P. (2016). Targeted Intron Retention and Excision for Rapid Gene Regulation in Response to Neuronal Activity. Neuron.

[14] Bai B., Wang X., Li Y., Chen P.C., Yu K., Dey K.K., Yarbro J.M., Han X., Lutz B.M., Rao S. (2020). Deep Multilayer Brain Proteomics Identifies Molecular Networks in Alzheimer’s Disease Progression. Neuron.

[15] Twine N.A., Janitz K., Wilkins M.R., Janitz M. (2011). Whole transcriptome sequencing reveals gene expression and splicing differences in brain regions affected by Alzheimer’s disease. PLoS ONE.

[16] Bai Y., Chen L., Zhang H. (2013). Intrοn retentiοn in SOD1 and GPX1 genes in Alzheimer’s disease. Free Radic. Biοl. Med..

[17] Middleton R., Gao D., Thomas A., Singh B., Au A., Wong J.J., Bomane A., Cosson B., Eyras E., Rasko J.E. (2017). IR Finder: Assessing the impact of intron retention on mammalian gene expression. Genome Biol..

[18] Boutz P.L., Bhutkar A., Sharp P.A. (2015). Detained introns are a novel, widespread class of post-transcriptionally spliced introns. Genes Dev..

[19] Naro C., Jolly A., Di Persio S., Bielli P., Setterblad N., Alberdi A.J., Vicini E., Geremia R., De la Grange P., Sette C. (2017). An orchestrated intron retention program in meiosis controls timely usage of transcripts during germ cell differentiation. Dev. Cell.

[20] Yap K., Lim Z.Q., Khandelia P., Friedman B., Makeyev E.V. (2012). Coordinated regulation of neuronal mRNA steady-state levels through developmentally controlled intron retention. Genes Dev..

[21] Buckley P.T., Khaladkar M., Kim J., Eberwine J. (2014). Cytoplasmic intron retention, function, splicing, and the sentinel RNA hypothesis. Wiley Interdiscip. Rev. RNA.

[22] Powers K.T., Szeto J.-Y.A., Schaffitzel C. (2020). New insights into no-go, non-stop and nonsense-mediated mRNA decay complexes. Curr. Opin. Struct. Biol..

[23] Lejeune F., Maquat L.E. (2005). Mechanistic links between nonsense-mediated mRNA decay and pre-mRNA splicing in mammalian cells. Curr. Opin. Cell Biol..

[24] Rekosh D., Hammarskjold M.L. (2018). Intron retention in viruses and cellular genes: Detention, border controls and passports. Wiley Interdiscip. Rev. RNA.

[25] Braunschweig U., Barbosa-Morais N.L., Pan Q., Nachman E.N., Alipanahi B., Gonatopoulos-Pournatzis T., Frey B., Irimia M., Blencowe B.J. (2014). Widespread intron retention in mammals functionally tunes transcriptomes. Genome Res..

[26] Wong J.J.-L., Au A.Y., Ritchie W., Rasko J.E. (2017). Intron retention in mRNA: No longer nonsense: Known and putative roles of intron retention innormal and disease biology. Bioessays.

[27] Mollet I.G., Ben-Dov C., Felício-Silva D., Grosso A.R., Eleutério P., Alves R., Staller R., Silva T.S., Carmo-Fonseca M. (2010). Unconstrained mining of transcript data reveals increased alternative splicing complexity in the human transcriptome. Nucleic Acids Res..

[28] Zheng J.T., Lin C.X., Fang Z.Y., Li H.D. (2020). Intron Retention as a Mode for RNA-Seq Data Analysis. Front. Genet..

[29] Jacob A.G., Smith C.W.J. (2017). Intron retention as a component of regulated gene expression programs. Hum. Genet..

[30] Grabski D.F., Broseus L., Kumari B., Rekosh D., Hammarskjold M.L., Ritchie W. (2021). Intron retention and its impact on gene expression and protein diversity: A review and a practical guide. Wiley Interdiscip. Rev. RNA.

[31] Buckley P.T., Lee M.T., Sul J.Y., Miyashiro K.Y., Bell T.J., Fisher S.A., Kim J., Eberwine J. (2011). Cytoplasmic intron sequence-retaining transcripts can be dendritically targeted via ID element retrotransposons. Neuron.

[32] Li H.D., Funk C.C., McFarland K., Dammer E.B., Allen M., Carrasquillo M.M., Levites Y., Chakrabarty P., Burgess J.D., Wang X. (2021). Integrative functional genomic analysis of intron retention in human and mouse brain with Alzheimer’s disease. Alzheimers Dement..

[33] Ping L., Duong D.M., Yin L., Gearing M., Lah J.J., Levey A.I., Seyfried N.T. (2018). Global quantitative analysis of the human brain proteome in Alzheimer’s and Parkinson’s disease. Sci. Data.

[34] Giannopoulou A.F., Konstantakou E.G., Velentzas A.D., Avgeris S.N., Avgeris M., Papandreou N.C., Zoi I., Filippa V., Katarachia S., Lampidonis A.D. (2019). Gene-Specific Intron Retention Serves as Molecular Signature that Distinguishes Melanoma from Non-Melanoma Cancer Cells in Greek Patients. Int. J. Mol. Sci..

[35] El-Seedy A., Pellerin L., Page G., Ladeveze V. (2023). Identification of Intron Retention in the Slc16a3 Gene Transcript Encoding the Transporter MCT4 in the Brain of Aged and Alzheimer-Disease Model (APPswePS1dE9) Mice. Genes.

[36] Ruiz-Gabarre D., Vallés-Saiz L., Carnero-Espejo A., Ferrer I., Hernández F., Garcia-Escudero R., Ávila J., García-Escudero V. (2024). Intron retention as a productive mechanism in human MAPT: RNA species generated by retention of intron 3. EBioMedicine.

[37] Dieter L.S., Estus S. (2010). Isoform of APOE with retained intron 3; quantitation and identification of an associated single nucleotide polymorphism. Mol. Neurodegener..

[38] Course M.M., Gudsnuk K., Keene C.D., Bird T.D., Jayadev S., Valdmanis P.N. (2023). Aberrant splicing of PSEN2, but not PSEN1, in individuals with sporadic Alzheimer’s disease. Brain.

[39] Casañas-Sánchez V., Pérez J.A., Fabelo N., Quinto-Alemany D., Díaz M.L. (2015). Docosahexaenoic (DHA) modulates phospholipid-hydroperoxide glutathione peroxidase (Gpx4) gene expression to ensure self-protection from oxidative damage in hippocampal cells. Front. Physiol..

[40] Han S., Nho K., Lee Y. (2020). Alternative Splicing Regulation of an Alzheimer’s Risk Variant in CLU. Int. J. Mol. Sci..

[41] Harold D., Abraham R., Hollingworth P., Sims R., Gerrish A., Hamshere M.L., Pahwa J.S., Moskvina V., Dowzell K., Williams A. (2009). Genome-wide association study identifies variants at CLU and PICALM associated with Alzheimer’s disease. Nat. Genet..

[42] Rockenstein E.M., McConlogue L., Tan H., Power M., Masliah E., Mucke L. (1995). Levels and alternative splicing of amyloid β protein precursor (APP) transcripts in brains of APP transgenic mice and humans with Alzheimer’s disease. J. Biol. Chem..

[43] Bu G. (2009). Apolipoprotein E and its receptors in Alzheimer’s Disease: Pathways, pathogenesis and therapy. Nat. Rev. Neurosci..

[44] Kim J., Basak J.M., Holtzman D.M. (2009). The role of Apolipoprotein E in Alzheimer’s Disease. Neuron.

[45] Boyles J.K., Pitas R.E., Wilson E., Mahley R.W., Taylor J.M. (1985). Apolipoprotein E associated with astrocytic glia of the CNS and with nonmyelinating glia of the peripheral nervous system. J. Clin. Investig..

[46] Pitas R.E., Boyles J.K., Lee S.H., Hui D., Weisgraber K.H. (1987). Lipoproteins and their receptors in the central nervous system. Characterization of the lipoproteins in cerebrospinal fluid and identification of apolipoprotein B,E(LDL) receptors in the brain. J. Biol. Chem..

[47] Grehan S., Tse E., Taylor J.M. (2001). Two distal downstream enhancers direct expression of the human apolipoprotein E gene to astrocytes in the brain. J. Neurosci..

[48] Stone D.J., Rozovsky I., Morgan T.E., Anderson C.P., Hajian H., Finch C.E. (1997). Astrocytes and microglia respond to estrogen with increased apoE mRNA in vivo and in vitro. Exp. Neurol..

[49] Xu Q., Walker D., Bernardo A., Brodbeck J., Balestra M.E., Huang Y. (2008). Intron-3 retention/splicing controls neuronal expression of apolipoprotein E in the CNS. J. Neurosc..

[50] Chen Z., Zhang D., Reynolds R.H., Gustavsson E.K., García-Ruiz S., D’Sa K., Fairbrother-Browne A., Vandrovcova J., Hardy J., International Parkinson’s Disease Genomics Consortium (IPDGC) (2021). Human-lineage-specific genomic elements are associated with neurodegenerative disease and APOE transcript usage. Nat. Commun..

[51] Muralidar S., Ambi S.V., Sekaran S., Thirumalai D., Palaniappan B. (2020). Role of tau protein in Alzheimer’s disease: The prime pathological player. Int. J. Biol. Macromol..

[52] Corsi A., Bombieri C., Valenti M.T., Romanelli M.G. (2022). Tau isoforms: Gaining insight into MAPT alternative splicing. Int. J. Mol. Sci..

[53] Andreadis A. (2005). Tau gene alternative splicing: Expression patterns, regulation and modulation of function in normal brain and neurodegenerative diseases. Biochim. Biophys. Acta Mol. Basis Dis..

[54] Liu F., Gong C.X. (2008). Tau exon 10 alternative splicing and tauopathies. Mol. Neurodegener..

[55] Wei M.L., Andreadis A. (1998). Splicing of a regulated exon reveals additional complexity in the axonal microtubule-associated protein tau. J. Neurochem..

[56] Gendron T.F., Petrucelli L. (2009). The role of tau in neurodegeneration. Mol. Neurodegener..

[57] Kosik K.S., Orecchio L.D., Bakalis S., Neve R.L. (1989). Developmentally regulated expression of specific tau sequences. Neuron.

[58] Amadoro G., Latina V., Corsetti V., Calissano P. (2020). N-terminal tau truncation in the pathogenesis of Alzheimer’s disease (AD): Developing a novel diagnostic and therapeutic approach. Biochim. Biophys. Acta Mol. Basis Dis..

[59] Reifert J., Hartung-Cranston D., Feinstein S.C. (2011). Amyloid beta-mediated cell death of cultured hippocampal neurons reveals extensive Tau fragmentation without increased full-length tau phosphorylation. J. Biol. Chem..

[60] Hsieh Y.C., Guo C., Yalamanchili H.K., Abreha M., Al-Ouran R., Li Y., Dammer E.B., Lah J.J., Levey A.I., Bennett D.A. (2019). Tau-Mediated Disruption of the Spliceosome Triggers Cryptic RNA Splicing and Neurodegeneration in Alzheimer’s Disease. Cell Rep..

[61] García-Escudero V., Ruiz-Gabarre D., Gargini R., Pérez M., García E., Cuadros R., Hernández I.H., Cabrera J.R., García-Escudero R., Lucas J.J. (2021). A new non-aggregative splicing isoform of human Tau is decreased in Alzheimer’s disease. Acta Neuropathol..

[62] Cuadros R., Pérez M., Ruiz-Gabarre D., Hernández F., García-Escudero V., Avila J. (2022). Specific Peptide from the Novel W-Tau Isoform Inhibits Tau and Amyloid β Peptide Aggregation In Vitro. ACS Chem. Neurosci..

[63] Ngian Z.K., Tan Y.Y., Choo C.T., Lin W.Q., Leow C.Y., Mah S.J., Lai M.K., Chen C.L., Ong C.T. (2022). Truncated Tau caused by intron retention is enriched in Alzheimer’s disease cortex and exhibits altered biochemical properties. Proc. Natl. Acad. Sci. USA.

[64] Domene-Serrano I., Cuadros R., Hernandez F., Avila J., Santa-Maria I. (2023). Tridimensional Structural Analysis of Tau Isoforms Generated by Intronic Retention. J. Alzheimers Dis. Rep..

[65] Ruiz-Gabarre D., Carnero-Espejo A., Ávila J., García-Escudero V. (2022). What’s in a Gene? The Outstanding Diversity of MAPT. Cells.

[66] García-Moreno J.F., Romão L. (2020). Perspective in Alternative Splicing Coupled to Nonsense-Mediated mRNA Decay. Int. J. Mol. Sci..

[67] Braggin J.E., Bucks S.A., Course M.M., Smith C.L., Sopher B., Osnis L., Shuey K.D., Domoto-Reilly K., Caso C., Kinoshita C. (2019). Alternative splicing in a presenilin 2 variant associated with Alzheimer disease. Ann. Clin. Transl. Neurol..

[68] Cheadle L., Biederer T. (2012). The novel synaptogenic protein Farp1 links postsynaptic cytoskeletal dynamics and transsynaptic organization. J. Cell Biol..

[69] Imai H., Nakagawa Y. (2003). Biological significance of phospholipid hydroperoxide glutathione peroxidase (PHGPx, GPx4) in mammalian cells. Free Radic. Biol. Med..

[70] Savaskan N.E., Ufer C., Kühn H., Borchert A. (2007). Molecular biology of glutathione peroxidase 4: From genomic structure to developmental expression and neural function. Biol. Chem..

[71] Ursini F., Maiorino M. (2020). Lipid peroxidation and ferroptosis: The role of GSH and GPx4. Free Radic. Biol. Med..

[72] Díaz M., Casañas-Sánchez V., Marín R., Pérez J.A., Catalá A. (2017). Fighting against Lipid Peroxidation in the Brain: The Unique Story of Docosahexaenoic Acid. Lipid Peroxidation: Inhibition, Effects and Mechanisms.

[73] Wong J.J., Ritchie W., Ebner O.A., Selbach M., Wong J.W., Huang Y., Gao D., Pinello N., Gonzalez M., Baidya K. (2013). Orchestrated intron retention regulates normal granulocyte differentiation. Cell.

[74] Liu G., Wang H., Liu J., Li J., Li H., Ma G., Jiang Y., Chen Z., Zhao B., Li K. (2014). The CLU gene rs11136000 variant is significantly associated with Alzheimer’s disease in Caucasian and Asian populations. Neuromol. Med..

[75] Zhang S., Li X., Ma G., Jiang Y., Liao M., Feng R., Zhang L., Liu J., Wang G., Zhao B. (2016). CLU rs9331888 Polymorphism Contributes to Alzheimer’s Disease Susceptibility in Caucasian But Not East Asian Populations. Mol. Neurobiol..

[76] Zhao L., Mao Z., Woody S.K., Brinton R.D. (2016). Sex differences in metabolic aging of the brain: Insights into female susceptibility to Alzheimer’s disease. Neurobiol. Aging.

[77] Schwartz S., Truglio M., Scott M.J., Fitzsimons H.L. (2016). Long-Term Memory in Drosophila Is Influenced by Histone Deacetylase HDAC4 Interacting with SUMO-Conjugating Enzyme Ubc9. Genetics.

[78] Miskalis A., Shirguppe S., Winter J., Elias G., Swami D., Nambiar A., Stilger M., Woods W.S., Gosstola N., Gapinske M. (2024). SPLICER: A highly efficient base editing toolbox that enables in vivo therapeutic exon skipping. Nat. Commun..

[79] Yuan H., Li N., Fu D., Ren J., Hui J., Peng J., Liu Y., Qiu T., Jiang M., Pan Q. (2017). Histone methyltransferase SETD2 modulates alternative splicing to inhibit intestinal tumorigenesis. J. Clin. Investig..

[80] Dvinge H., Bradley R.K. (2015). Widespread intron retention diversifies most cancertranscriptomes. Genome Med..

[81] Bhadra M., Howell P., Dutta S., Heintz C., Mair W.B. (2020). Alternative splicing in aging and longevity. Hum. Genet..

[82] Ong C.T., Adusumalli S. (2020). Increased intron retention is linked to Alzheimer’s disease. Neural Regen. Res..

[83] Valadkhan S., Mohammadi A., Wachtel C., Manley J.L. (2007). Protein-free spliceosomal snRNAs catalyze a reaction that resembles the first step of splicing. RNA.

[84] Valadkhan S., Manley J.L. (2001). Splicing-related catalysis by protein-free snRNAS. Nature.

[85] Valadkhan S., Mohammadi A., Jaladat Y., Geisler S. (2009). Protein-free small nuclear RNAs catalyze a two-step splicing reaction. Proc. Natl. Acad. Sci. USA.

[86] Hu L.F., Li Y.X., Wang J.Z., Zhao Y.T., Wang Y. (2023). Controlling CRISPR-Cas9 by guide RNA engineering. Wiley Interdiscip. Rev. RNA.

[87] Sznajder L.J., Thomas J.D., Carrell E.M., Reid T., McFarland K.N., Cleary J.D., Oliveira R., Nutter C.A., Bhatt K., Sobczak K. (2018). Intron retention induced by microsatellite expansions as a disease biomarker. Proc. Natl. Acad. Sci. USA.

[88] Vanichkina D.P., Schmitz U., Wong J.J., Rasko J.E.J. (2018). Challenges in defining the role of intron retention in normal biology and disease. Semin. Cell Dev. Biol..

